# Late Stage at Diagnosis of Cervical Cancer and Its Correlates at a Large Regional Referral Hospital in Uganda: A Cross-Sectional Study

**DOI:** 10.7759/cureus.62702

**Published:** 2024-06-19

**Authors:** Rogers Kajabwangu, Francis Bajunirwe, Jonathan Izudi, Joel Bazira, Frank Ssedyabane, Musa Kayondo, Henry M Lugobe, Stuart Turanzomwe, Thomas C Randall, Joseph Ngonzi

**Affiliations:** 1 Department of Obstetrics and Gynaecology, Mbarara University of Science and Technology, Mbarara, UGA; 2 Department of Community Health, Mbarara University of Science and Technology, Mbarara, UGA; 3 Department of Microbiology, Mbarara University of Science and Technology, Mbarara, UGA; 4 Department of Medical Laboratory Science, Mbarara University of Science and Technology, Mbarara, UGA; 5 Obstetrics, Gynecology and Reproductive Biology, Harvard Medical School, Boston, USA

**Keywords:** diagnosis, correlates, uganda, late stage, cervical cancer

## Abstract

Background

The stage of disease at diagnosis is one of the major determinants of survival in women with cervical cancer. Most women with cervical cancer in low- and middle-income countries (LMICs) present to hospitals with advanced stages, thus reducing their survivorship following the diagnosis. Factors correlated with late-stage disease at diagnosis are not completely explored. This study aimed to describe the association between sociodemographic, clinical, and metabolic characteristics with late-stage disease at diagnosis in women with cervical cancer attending the Mbarara Regional Referral Hospital in Southwestern Uganda.

Methodology

We conducted a cross-sectional study of women with histological diagnoses of invasive cervical cancer between November 2022 and August 2023. Women who presented to the hospital with the International Federation of Gynecology and Obstetrics stage IIb and above were considered to have late-stage cervical cancer while those with stage IIa and below were considered to have early-stage disease. We used modified Poisson regression to determine the factors independently associated with the outcome.

Results

We enrolled 157 women. The average age of the participants was 52.4 years. The majority of the participants (83.4%) had late-stage disease at diagnosis. Women with adenocarcinoma (adjusted prevalence ratio (aPR) = 1.18, 95% confidence interval (CI) = 1.10-1.38) and those with lymphovascular space involvement on histology (aPR = 1.30, 95% CI = 1.05-1.60) were more likely to have late-stage disease at diagnosis while women living with human immunodeficiency virus (aPR = 0.83, 95% CI = 0.71-0.97) were less likely to present with late-stage disease at diagnosis. None of the sociodemographic and metabolic characteristics were associated with late-stage disease at diagnosis.

Conclusions

The number of women presenting with late-stage cervical cancer is high. Efforts to increase the availability and uptake of cervical cancer screening services in LMICs should be reinforced. Cervical cancer treatment services should be decentralized to increase accessibility.

## Introduction

The incidence of cervical cancer in Sub-Saharan Africa continues to rise, with some countries reporting as many as 85 cases per 100,000 women-years [1]. This is predominantly attributed to inadequate cervical cancer screening services [2], leading to a very low screening uptake rate [3]. With over 36,000 deaths in 2020, the East African region reported the highest mortality rate from cervical cancer in the world [1]. The healthcare systems in these countries are challenged by inadequate diagnostic facilities and insufficient access to standard treatment for cervical cancer [4]. In Uganda, for example, there is only one center that provides radiotherapy services to the entire population which is located in the capital of the country [5]. This means that patients from other parts of the country have to travel long distances to reach the center and some are completely unable to access the treatment. Consequently, the survival rate for cervical cancer patients in low- and middle-income countries (LMICs) is very low [6].

The stage of disease at presentation has been identified as one of the major factors that influence survival among cervical cancer patients [7]. Existing data show that most women with cervical cancer in LMICs present with advanced stages of the disease [8]. However, context-specific data on the factors that influence stage at presentation in cervical cancer patients is scarce. The two studies conducted in Uganda by Wu et al. and Mwaka et al. only focused on a limited range of factors [9,10].

Therefore, this study aimed to describe a broad range of factors including sociodemographic, clinical, and metabolic risk factors associated with late-stage cervical cancer disease at diagnosis in women in Southwestern Uganda.

## Materials and methods

Study design, setting, and population

This was a cross-sectional study of women with histological diagnoses of invasive cervical cancer at Mbarara Regional Referral Hospital (MRRH) between November 2022 and August 2023. MRRH is a tertiary hospital in Southwestern Uganda serving 13 districts with a population of approximately 4 million people. It also provides services to parts of Rwanda, Burundi, Tanzania, and the Democratic Republic of Congo, which are neighboring Uganda. The cervical cancer screening clinic attends to an average of 15 women per day and operates five days a week. Health workers at the clinic include several nursing officers, residents, and gynecologists. The cervical cancer screening tests at the clinic include visual inspection methods, colposcopy, and conventional cytology. Patients with suspicious lesions for cervical cancer undergo cervical biopsy followed by histopathological analysis for definitive diagnosis. On average, 10 women are diagnosed with cervical cancer per month. Patients with confirmed cervical cancer are transferred to the gynecology ward for clinical staging and surgical intervention for early-stage cases (≤2A). Those with advanced cancer are referred to the Uganda Cancer Institute at Mulago National Referral Hospital for chemoradiotherapy.

Ethical approval for the study was obtained from the Mbarara University of Science and Technology Research Ethics Committee (MUST-2022-576) and the National Council for Science and Technology (HS3053ES). Informed consent was obtained from all participants.

Measurements

The outcome variable was late-stage disease at diagnosis. Staging was done clinically and cervical cancer patients with stage IIb and above based on the International Federation of Obstetrics and Gynecology staging system [11] were considered as having a late-stage invasive cervical cancer diagnosis while those with stage IIa and below were considered to have early-stage disease.

The independent variables included sociodemographic data such as age, level of education, residence, distance from hospital, marital status, parity, and age at sexual debut. Clinical data included a history of cervical cancer screening, lifetime sexual partners, human immunodeficiency virus (HIV) infection, hypertension, hyperglycemia, waist circumference, high-density lipoprotein level, serum triglycerides, and metabolic syndrome which was defined using the National Cholesterol Education Programme Adult Treatment Panel III criteria as the presence of three or more of the following criteria [12]: central obesity, defined as waist circumference >88 cm, elevated triglycerides (≥150 mg/dL or 1.695 mmol/L), low high-density lipoprotein (<50 mg/dL, equivalent to <1.295 mmol/L), high blood pressure (systolic blood pressure ≥130 mmHg or diastolic blood pressure ≥85 mmHg), and high fasting blood glucose (≥100 mg/dL or 5.6 mmol/L) [12].

Waist circumference was measured to the nearest 0.1 cm using a measuring tape in the horizontal plane midway between the inferior margin of the ribs and the superior border of the iliac crest, with the measurement recorded at minimal respiration. Blood pressure was measured after five minutes of rest on the left arm in mmHg by the auscultation method using a calibrated sphygmomanometer and a stethoscope. Three readings were taken at a five-minute interval and the average of the last two systolic and diastolic measurements was taken as the mean systolic blood pressure and diastolic blood pressure, respectively. Fasting blood sugar was measured using a glucometer after at least eight hours of fasting using capillary blood. Blood was then collected for fasting triglycerides and high-density lipoprotein as follows: 4 mL of venous blood was aseptically drawn from the mid-cubital vein by venepuncture and collected into plain vacutainers. Each specimen was labeled with a unique identification number and left to clot at room temperature for two hours. Subsequently, the specimens were transported to the laboratory, where centrifugation was performed at 1,000× g for 15 minutes at 2~8℃ to separate serum from blood cells. The resultant serum was then carefully transferred into cryovial tubes using a micropipette. Fasting triglyceride and high-density lipoprotein concentrations were measured using a fully automated analyzer (Cobas 6000 Clinical Chemistry Analyzer; Roche Diagnostics International, Rotkreuz, Switzerland). Histological data included histology, lymphovascular invasion, and level of differentiation.

Statistical analysis

The data analysis was performed in Stata version 15 (StataCorp., College Station, TX, USA). In the univariate analysis, we summarized categorical data using frequencies and proportions, and numerical data using means and standard deviations. In the bivariate analysis, we used the chi-square test to test differences in the proportions of categorical variables with the outcome variable when the cell count was large (five or more) and the Fisher’s exact test when the cell count was small (less than five). We tested mean differences in numerical data with the outcome variable using the Student’s t-test. In the multivariable analysis, we used the modified Poisson regression analysis with robust standard errors to determine factors independently associated with the outcome reported as prevalence ratio (PR) and 95% confidence interval (CI). In the multivariable analysis, variables with a p-value <0.2 on bivariate analysis and those with biologic plausibility were included. The PR was preferred over the odds ratio (OR) as the outcome was large and using the OR would overestimate the degree of association compared [13].

## Results

Characteristics of the participants

A total of 157 women were enrolled in the study. The average age of the participants was 52.4 years (standard deviation, 13.1), as shown in Table 1. The proportion of the participants with late-stage disease at diagnosis was 83.4% (95% CI = 76.7-88.5). Participants with early-stage disease at diagnosis were similar to those with late-stage disease at diagnosis regarding almost all sociodemographic, clinical, and histological characteristics except lymphovascular space involvement (p = 0.037). However, HIV infection was more prevalent among patients with early-stage disease compared to those with late-stage disease (61.5% vs. 35.9%, p = 0.015).

**Table 1:** Participant characteristics. SD = standard deviation; HIV = human immunodeficiency virus

Variable	Category	Overall	Late stage	Early stage	P-value
		(N = 157)	(n = 131)	(n = 26)	
Age	Mean (SD)	52.4 (13.1)	53.2 (13.5)	48.3 (10.4)	0.082
Age category	<50 years	65 (41.4)	52 (39.7)	13 (50.0)	0.330
	≥50 years	92 (58.6)	79 (60.3)	13 (50.0)	
Level of education	No formal education	62 (39.5)	53 (40.5)	9 (34.6)	0.520
	Primary education	80 (51.0)	67 (51.1)	13 (50.0)	
	Secondary and above	15 (9.6)	11 (8.4)	4 (15.4)	
Residence	Urban	8 (5.1)	7 (5.3)	1 (3.8)	0.850
	Semi-urban	9 (5.7)	8 (6.1)	1 (3.8)	
	Rural	140 (89.2)	116 (88.5)	24 (92.3)	
Distance from hospital (km)	≤10	2(1.3)	1(3.8)	1(0.8)	0.200
	>10	155 (98.7)	25 (96.2)	130 (99.2)	
Marital status	Married	83 (52.9)	71 (54.2)	12 (46.2)	0.521
	Separated/Divorced	39 (24.8)	33 (25.2)	6 (23.1)	
	Widowed	35 (22.3)	27 (20.6)	8 (30.8)	
Parity	Nulliparous	8 (5.1)	5 (3.8)	3 (11.5)	0.162
	Multiparous	39 (24.8)	31 (23.7)	8 (30.8)	
	Grand-multiparous	110 (70.1)	95 (72.5)	15 (57.7)	
Age at sexual debut in years	≤16	60 (38.2)	51 (38.9)	9 (34.6)	0.691
	17–20	75 (47.8)	63 (48.1)	12 (46.2)	
	≥21	22 (14.0)	17 (13.0)	5 (19.2)	
	No	70 (44.6)	62 (47.3)	8 (30.8)	
Number of lifetime sexual partners	1	72 (45.9)	62 (47.3)	10 (38.5)	0.411
	≥2	85 (54.1)	69 (52.7)	16 (61.5)	
HIV infection	No	94 (59.9)	84 (64.1)	10 (38.5)	0.015
	Yes	63 (40.1)	47 (35.9)	16 (61.5)	
Waist circumference	Normal	105 (66.9)	19 (73.1)	86 (65.6)	0.460
	Central obesity	52 (33.1)	7 (26.9)	45 (34.4)	
High-density lipoprotein	Normal	26 (16.6)	3 (11.5)	23 (17.6)	0.451
	Low	131 (83.4)	23 (88.5)	108 (82.4)	
Serum triglycerides	Normal triglycerides	122 (77.7)	22 (84.6)	100 (76.3)	0.350
	Hypertriglyceridemia	35 (22.3)	4 (15.4)	31 (23.7)	
Hypertension	No	72 (45.9)	12 (46.2)	60 (45.8)	0.970
	Yes	85 (54.1)	14 (53.8)	71 (54.2)	
Hyperglycemia	No	29 (18.5)	5 (19.2)	24 (18.3)	0.911
	Yes	128 (81.5)	21 (80.8)	107 (81.7)	
Metabolic syndrome	Absent	66 (42.0)	11 (42.3)	55 (42.0)	0.981
	Present	91 (58.0)	15 (57.7)	76 (58.0)	
Histological type cervical cancer	Squamous cell carcinoma	141 (89.8)	116 (88.5)	25 (96.2)	0.241
	Adenocarcinoma	16 (10.2)	15 (11.5)	1 (3.8)	
Lymphovascular invasion	Absent	38 (24.2)	27 (20.6)	11 (42.3)	0.037
	Present	64 (40.8)	58 (44.3)	6 (23.1)	
	Not reported	55 (35.0)	46 (35.1)	9 (34.6)	
Level of differentiation	Well-differentiated	42 (26.8)	32 (24.4)	10 (38.5)	0.260
	Moderately differentiated	32 (20.4)	28 (21.4)	4 (15.4)	
	Poorly differentiated	83 (52.9)	71 (54.2)	12 (46.2)	

On bivariate analysis, adenocarcinoma and lymphovascular space involvement on histology were associated with late-stage disease at diagnosis while HIV-positive status was protective. Age, residence type, and distance from the hospital were considered in the multivariate analysis due to their biologic plausibility. On adjusted analysis, patients with adenocarcinoma and those with lymphovascular space involvement on histology were more likely to present with late-stage cervical cancer while those living with HIV were less likely to present with late-stage disease at diagnosis. None of the sociodemographic and metabolic factors was associated with late stage at diagnosis (Table 2).

**Table 2:** Factors associated with late-stage diagnosis. cPR = crude prevalence ratio; aPR = adjusted prevalence ratio; CI = confidence interval; HIV = human immunodeficiency virus; HDL = high-density lipoprotein

Characteristics	Level	Modified Poisson regression analysis			
		Unadjusted analysis		Adjusted analysis	
		cPR	95% CI	aPR	95% CI
Age category	<50 years	1		1	
	≥50 years	1.07	0.92-1.24	1.03	0.90-1.18
Distance(km)	≤10			1	
	>10	1.67	0.42-6.75	1.60	0.39-6.56
Residence type	Urban	1		1	
	Semi-urban	1.02	0.71-1.44	0.94	0.68-1.29
	Rural	0.95	0.72-1.24	0.85	0.65-1.10
Level of education	Secondary and above	1			
	Primary education	1.14	0.83-1.57	-	-
	No formal education	1.17	0.84-1.61	-	-
Marital status	Married	1			
	Separated/Divorced	0.99	0.84-1.61	-	-
	Widowed	0.90	0.74-1.10	-	-
Parity	Nulliparous	1			
	Multiparous	1.27	0.73-2.23	-	-
	Grand-multiparous	1.38	0.80-1.07	-	-
Age at sexual debut	≥21 years	1			
	17–20 years	1.09	0.85-1.39	-	-
	≤16 years	1.10	0.85-1.41	-	-
Sexual partners in life	1	1			
	≥2	0.94	0.82-1.08	-	
HIV infection	No	1		1	
	Yes	0.83	0.71-0.98	0.83	0.71-0.97
Hypertension	No	1			
	Yes	1.00	0.87-1.15	-	-
Hyperglycemia	No	1			
	Yes	1.01	0.84-1.21	-	-
Waist circumference	Normal	1			
	Central obesity	1.06	0.92-1.22	-	-
HDL	Normal	1			
	Low	0.93	0.79-1.09	-	-
Serum triglycerides	Normal	1			
	Elevated	1.08	0.93-1.25	-	-
Metabolic syndrome	Absent	1			
	Present	1.00	0.87-1.15	-	-
Histology	Squamous cell carcinoma	1			
	Adenocarcinoma	1.14	0.98-1.32	1.18	1.01-1.38
Lymphovascular invasion	Absent	1		1	
	Present	1.28	1.02-1.59	1.30	1.05-1.60
	Not reported	1.17	0.93-1.49	1.16	0.92-1.45
Level of differentiation	Well-differentiated	1		1	
	Moderately differentiated	1.12	0.94-1.40	1.12	091-1.39
	Poorly differentiated	1.31	0.95-1.35	1.12	0.92-1.36

## Discussion

In this study, we describe the burden of late-stage disease at diagnosis of cervical cancer among women in Southwestern Uganda and its association with the sociodemographic, clinical, and metabolic characteristics of the women. We found that more than 8 in 10 women with cervical cancer present with late-stage disease at diagnosis. Late stage at diagnosis was more common among women who had adenocarcinoma histology and lymphovascular space involvement. In contrast, women living with HIV were less likely to present with late stage at diagnosis.

The high proportion of cervical cancer patients presenting with late stage at diagnosis presents challenges with the management and treatment. Previous studies in Uganda and other low-resource settings have shown similar results [9,14]. This is likely a result of low screening uptake due to inadequate screening services in addition to the numerous infrastructural and human resource challenges in the cervical cancer care pathway [5,15]. As the stage at presentation is a major determinant of prognosis in cervical cancer [16], it is important that the cancer is diagnosed and treated at an early stage. This implies that screening programs must be improved and decentralized centers established to improve accessibility to cervical cancer treatment.

Compared with those with squamous cell carcinoma, women with adenocarcinoma were more likely to have late-stage disease at diagnosis. The late stage at diagnosis in adenocarcinoma may be explained by the difficulty in diagnosing it as the initial growth is within the cervical canal [17]. The lesions are therefore easily missed during cytology of brushings from the ectocervix, or with colposcopy and cervical biopsy [18]. Human papillomavirus (HPV)-based screening would ideally solve this challenge by enabling diagnosis of all precancer lesions regardless of histology. Although there is a recommendation by the World Health Organization for cervical cancer screening programs to predominantly use HPV DNA testing [19], many low-resource settings are still using mainly visual inspection methods and cytology for cervical cancer screening [5] as the facilities for HPV testing are either unavailable or inadequate [20].

Lymphovascular space involvement was independently associated with the late stage of cervical cancer at diagnosis. This finding is supportive of prior observations of poor prognosis in cervical cancer patients with lymphovascular space involvement. A Gynaecologic Oncologic Group study found a higher rate of recurrence of cervical cancer in the presence of lymphovascular space involvement [21]. Jena and colleagues also found lymphovascular space involvement to increase the risk of recurrence in the presence of angiovascular involvement [22].

Compared to women not living with HIV, women living with HIV infection in this study were less likely to present with late-stage disease. This may be partly explained by the fact that in Uganda and several other high HIV burden settings, people with HIV infection are now on antiretroviral therapy [23] and are more likely to undergo regular and routine screening for cervical cancer. The services for cervical cancer screening have largely been successfully incorporated into the HIV care programs [24]. Although HIV infection has been found to increase the risk of cervical cancer [25], several studies have found no association between the infection and the stage of cervical cancer at diagnosis [10,26].

Demographic factors such as age, marital status, education level, and distance of the participants’ residence from the hospital were not associated with the stage at presentation in this study, a finding that is similar to those of previous studies [9,27,28]. Metabolic syndrome and its individual components, i.e., hypertension, hyperglycemia, central obesity, low high-density lipoprotein level, and hypertriglyceridemia, were not associated with late-stage diagnosis in this study. Although data on the effect of metabolic syndrome and its components on the stage of cervical cancer at presentation is scarce, several studies have indicated poorer prognosis, as evidenced by higher rates of recurrence and mortality in cervical cancer patients with metabolic syndrome or its individual components [29,30].

Our study has some strengths and limitations which should be considered in the interpretation of the results. First, as the participants were prospectively recruited, we were able to obtain data on all the variables required to complete the analysis. Second, the diagnosis of cervical cancer was based on histological examination with adequate detail. However, the histopathological classification was limited to the two major groups of squamous cell carcinoma and adenocarcinoma without further sub-classification. In addition, the study had a disproportionately high number of patients with late-stage cancer and very few patients with early-stage cancer. Although it was important to establish the distribution of early versus late diagnosis, the high prevalence of the outcome posed a challenge in the regression analysis. We addressed this limitation by using Poisson regression analysis to make conclusions on association. Future studies can be designed to have comparable numbers of patients with early and late-stage cancer. Finally, as our study was conducted at a single tertiary hospital, the findings may not be generalizable to the entire population of Ugandan women with cervical cancer.

## Conclusions

We found a very high rate of late-stage disease among cervical cancer patients at MRRH. Adenocarcinoma histology and lymphovascular space involvement were associated with late stage at diagnosis while HIV-infected patients were less likely to present with late stage at diagnosis. Efforts to increase the availability and uptake of HPV-based cervical cancer screening services in LMICs should be reinforced. Cervical cancer treatment services should be decentralized to increase accessibility.

## References

[1] Singh D, Vignat J, Lorenzoni V (2023). Global estimates of incidence and mortality of cervical cancer in 2020: a baseline analysis of the WHO Global Cervical Cancer Elimination Initiative. Lancet Glob Health.

[2] Huh WK, Ault KA, Chelmow D (2015). Use of primary high-risk human papillomavirus testing for cervical cancer screening: interim clinical guidance. J Low Genit Tract Dis.

[3] Ndejjo R, Mukama T, Musabyimana A, Musoke D (2016). Uptake of cervical cancer screening and associated factors among women in rural Uganda: a cross sectional study. PLoS One.

[4] Swanson M, Ueda S, Chen LM, Huchko MJ, Nakisige C, Namugga J (2018). Evidence-based improvisation: facing the challenges of cervical cancer care in Uganda. Gynecol Oncol Rep.

[5] Nakisige C, Schwartz M, Ndira AO (2017). Cervical cancer screening and treatment in Uganda. Gynecol Oncol Rep.

[6] Gondos A, Brenner H, Wabinga H, Parkin DM (2005). Cancer survival in Kampala, Uganda. Br J Cancer.

[7] Sengayi-Muchengeti M, Joko-Fru WY, Miranda-Filho A (2020). Cervical cancer survival in sub-Saharan Africa by age, stage at diagnosis and Human Development Index: a population-based registry study. Int J Cancer.

[8] Stewart TS, Moodley J, Walter FM (2018). Population risk factors for late-stage presentation of cervical cancer in sub-Saharan Africa. Cancer Epidemiol.

[9] Mwaka AD, Garimoi CO, Were EM, Roland M, Wabinga H, Lyratzopoulos G (2016). Social, demographic and healthcare factors associated with stage at diagnosis of cervical cancer: cross-sectional study in a tertiary hospital in Northern Uganda. BMJ Open.

[10] Wu ES, Urban RR, Krantz EM (2020). The association between HIV infection and cervical cancer presentation and survival in Uganda. Gynecol Oncol Rep.

[11] Shi J, Dong Y, Jiang W (2022). MRI-based peritumoral radiomics analysis for preoperative prediction of lymph node metastasis in early-stage cervical cancer: a multi-center study. Magn Reson Imaging.

[12] Grundy SM, Cleeman JI, Daniels SR (2005). Diagnosis and management of the metabolic syndrome: an American Heart Association/National Heart, Lung, and Blood Institute Scientific Statement. Circulation.

[13] Spiegelman D, Hertzmark E (2005). Easy SAS calculations for risk or prevalence ratios and differences. Am J Epidemiol.

[14] Dereje N, Gebremariam A, Addissie A (2020). Factors associated with advanced stage at diagnosis of cervical cancer in Addis Ababa, Ethiopia: a population-based study. BMJ Open.

[15] Moodley J, Kawonga M, Bradley J, Hoffman M (2006). Challenges in implementing a cervical screening program in South Africa. Cancer Detect Prev.

[16] Balasubramaniam G, Gaidhani RH, Khan A, Saoba S, Mahantshetty U, Maheshwari A (2021). Survival rate of cervical cancer from a study conducted in India. Indian J Med Sci.

[17] Gunnell AS, Ylitalo N, Sandin S, Sparén P, Adami HO, Ripatti S (2007). A longitudinal Swedish study on screening for squamous cell carcinoma and adenocarcinoma: evidence of effectiveness and overtreatment. Cancer Epidemiol Biomarkers Prev.

[18] Gien LT, Beauchemin MC, Thomas G (2010). Adenocarcinoma: a unique cervical cancer. Gynecol Oncol.

[19] Gultekin M, Ramirez PT, Broutet N, Hutubessy R (2020). World Health Organization call for action to eliminate cervical cancer globally. Int J Gynecol Cancer.

[20] Fokom Domgue J, Futuh B, Ngalla C (2020). Feasibility of a community-based cervical cancer screening with "test and treat" strategy using self-sample for an HPV test: experience from rural Cameroon, Africa. Int J Cancer.

[21] Delgado G, Bundy B, Zaino R, Sevin BU, Creasman WT, Major F (1990). Prospective surgical-pathological study of disease-free interval in patients with stage IB squamous cell carcinoma of the cervix: a Gynecologic Oncology Group study. Gynecol Oncol.

[22] Hertel H, Köhler C, Michels W, Possover M, Tozzi R, Schneider A (2003). Laparoscopic-assisted radical vaginal hysterectomy (LARVH): prospective evaluation of 200 patients with cervical cancer. Gynecol Oncol.

[23] Babatunde AO, Akin-Ajani OD, Abdullateef RO, Togunwa TO, Isah HO (2023). Review of antiretroviral therapy coverage in 10 highest burden HIV countries in Africa: 2015-2020. J Med Virol.

[24] Castle PE, Einstein MH, Sahasrabuddhe VV (2021). Cervical cancer prevention and control in women living with human immunodeficiency virus. CA Cancer J Clin.

[25] Abraham AG, D'Souza G, Jing Y (2013). Invasive cervical cancer risk among HIV-infected women: a North American multicohort collaboration prospective study. J Acquir Immune Defic Syndr.

[26] Moodley JR, Hoffman M, Carrara H (2006). HIV and pre-neoplastic and neoplastic lesions of the cervix in South Africa: a case-control study. BMC Cancer.

[27] Dunyo P, Effah K, Udofia EA (2018). Factors associated with late presentation of cervical cancer cases at a district hospital: a retrospective study. BMC Public Health.

[28] Appiah-Kubi A, Konney TO, Amo-Antwi K (2022). Factors associated with late-stage presentation of cervical cancer in Ghana. Ghana Med J.

[29] Ahn HK, Shin JW, Ahn HY, Park CY, Lee NW, Lee JK, Hwang IC (2015). Metabolic components and recurrence in early-stage cervical cancer. Tumour Biol.

[30] Jee SH, Ohrr H, Sull JW, Yun JE, Ji M, Samet JM (2005). Fasting serum glucose level and cancer risk in Korean men and women. JAMA.

